# Physiological Functions of Side-Chain-Retaining Sterols in the Brain and Their Roles in Neurodegenerative Diseases

**DOI:** 10.3390/metabo16030189

**Published:** 2026-03-11

**Authors:** Yoshimitsu Kiriyama, Akira Nakatsuma, Hiroshi Tokumaru, Hisayo Sadamoto, Hiromi Nochi

**Affiliations:** 1 Kagawa School of Pharmaceutical Sciences, Tokushima Bunri University, Hamanochou 8-53, Takamatsu 760-8542, Kagawa, Japan; nakatsumaa@kph.bunri-u.ac.jp (A.N.); tokumaruh@kph.bunri-u.ac.jp (H.T.); sadamotoh@kph.bunri-u.ac.jp (H.S.); nochi@kph.bunri-u.ac.jp (H.N.); 2 Institute of Neuroscience, Tokushima Bunri University, Hamanochou 8-53, Takamatsu 760-8542, Kagawa, Japan

**Keywords:** cholesterol, desmosterol, 24S-hydroxycholesterol, CDCA, LXR, neurogenerative diseases, Alzheimer’s disease, Parkinson’s disease, Huntington’s disease, multiple sclerosis

## Abstract

Although the brain comprises only 2% of total body weight, it contains approximately 23% of the total cholesterol of the body. In the brain, cholesterol plays a critical role as a structural component of cell membranes and myelin sheaths. However, the blood–brain barrier restricts cholesterol influx from the systemic circulation into the brain. As a result, the brain synthesizes cholesterol de novo and regulates its metabolism independently. Desmosterol, a cholesterol precursor produced during cholesterol biosynthesis, and cholesterol metabolites, 24S-hydroxycholesterol and chenodeoxycholic acid, are sterols with structurally retained side chains. These side-chain-retaining sterols have traditionally been regarded as intermediates in the cholesterol synthesis process or as metabolites for cholesterol excretion, but accumulating evidence indicates that they also function as physiologically active signaling molecules that influence brain function via nuclear receptors, such as liver X receptors, and membrane receptors, such as NMDA receptors. Through nuclear receptors, these side-chain-retaining sterols regulate the transcription of genes involved in lipid transport, inflammation control, and amyloid clearance, while their membrane receptor action enables rapid synaptic effects. These side-chain-retaining sterols mediate metabolic crosstalk between neurons and glial cells and contribute to maintaining cholesterol balance in the developing brain. Furthermore, these side-chain-retaining sterols have been shown to affect amyloid-β clearance, α-synuclein aggregation, neuroinflammation, mitochondrial function, and remyelination. Dysregulation of these side-chain-retaining sterols is associated with neurodegenerative diseases such as Alzheimer’s disease and Parkinson’s disease. Overall, side-chain-retaining sterols are important regulators of brain physiology. This review focuses on the current knowledge regarding the physiological functions of side-chain-retaining sterols in the brain and their roles in neurodegenerative diseases.

## 1. Introduction

Lipids represent a fundamental structural component of the brain. They account for approximately 50% of the brain’s dry weight; thus, the brain ranks as the second most lipid-rich tissue in the human body, surpassed only by adipose tissue [1]. However, unlike adipose tissue, where lipids primarily function as an energy reservoir, the dense lipid composition of the brain is essential for maintaining the complex structural framework required for neuronal function. Brain lipids include cholesterol, phospholipids, such as phosphatidylcholine and phosphatidylethanolamine, and sphingolipids, such as ceramide [1,2,3,4]. Among these lipid classes, the brain contains a higher proportion of cholesterol than other organs. Although it accounts for only 2% of total body weight, the brain contains approximately 23% of the body’s total cholesterol [5]. In the brain, cholesterol predominantly exists in an unesterified form, which facilitates its rapid incorporation into cell membranes and synaptic structures [5,6,7,8]. One defining biological feature of brain-derived cholesterol is its metabolic autonomy. The blood–brain barrier (BBB) is a tightly regulated boundary that effectively prevents cholesterol transfer from the systemic circulation into the brain. This physical separation requires the brain to maintain its own cholesterol synthesis and metabolism. Therefore, most brain cholesterol is produced through de novo synthesis within the brain, and its metabolism is regulated independently of peripheral tissues [5]. This autonomy indicates that cholesterol metabolism in the brain is largely uncoupled from systemic cholesterol homeostasis in peripheral organs, such as the liver. In addition, the high abundance of cholesterol and its related molecules in the brain likely reflects their critical roles in brain function. Cholesterol stabilizes myelin sheaths, thereby facilitating rapid axonal signal transmission, and maintains the fluidity of neuronal membranes. However, cholesterol is not solely an inert structural component, as it also functions as a signaling molecule. Cholesterol plays an important role in brain activity and in neurodegenerative diseases [9,10,11].

As the diverse physiological and pathological effects of cholesterol in the brain are increasingly understood, the roles of cholesterol biosynthetic intermediates and metabolites are beginning to be recognized. The cholesterol precursor, desmosterol, and cholesterol metabolites, 24S-hydroxycholesterol (24S-HC) and chenodeoxycholic acid (CDCA), are sterols with structurally retained side chains. These side-chain-retaining sterols were previously considered intermediates in cholesterol synthesis or metabolites for cholesterol excretion. However, accumulating evidence demonstrates that they function as physiologically active signaling molecules that act as ligands for receptors, such as liver X receptor (LXR) or farnesoid X receptor (FXR). Furthermore, these sterols have been implicated in neurodegenerative diseases, including Alzheimer’s disease, Parkinson’s disease, and Huntington’s disease. Unlike cholesterol, 24S-hydroxycholesterol and chenodeoxycholic acid possess physicochemical properties that allow them to cross the BBB [12,13]. Thus, the unique permeability of these side-chain-retaining sterols suggests that peripherally derived cholesterol-related substances can influence neurodegenerative disease states and may represent effective therapeutic routes.

Brain lipid metabolism encompasses major lipid classes, including cholesterol, phospholipids, and sphingolipids, which collectively contribute to cell membrane construction and myelination. These lipid classes do not function independently but instead interact to regulate the brain membrane environment and cellular responses. In particular, side-chain-retaining sterols may influence membrane lipid composition by regulating the expression of lipid transporters and phospholipid metabolism-related genes through transcriptional control mediated by nuclear receptors, such as LXR [14,15]. Furthermore, cholesterol and sphingolipids cooperatively participate in lipid raft formation, thereby determining receptor localization and signal transduction efficiency [16]. Consequently, the physiological effects of side-chain-retaining sterols mediated by membrane receptors are likely expressed through crosstalk with other lipid classes. While phospholipids and sphingolipids primarily support membrane structure, cholesterol and its metabolites play distinct roles in modulating membrane properties and functioning as receptor ligands. The findings discussed in this review are supported by evidence from multiple experimental approaches, including studies using cultured cells and animal models, rather than relying on a single experimental system. This review focuses on side-chain-retaining sterols, which are particularly prominent as important signaling molecules, and outlines their physiological roles and implications for major neurodegenerative diseases.

## 2. Biosynthesis of Cholesterol Precursors and Side-Chain Retaining Sterols in the Brain

### 2.1. Biosynthesis and Transport of Cholesterol Precursors and Cholesterol in the Brain

**Figure 1:**
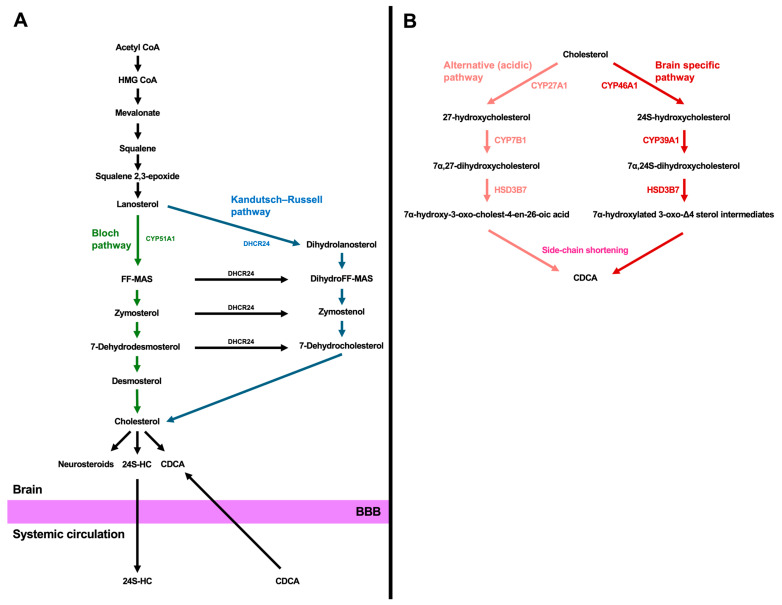
(**A**) Cholesterol biosynthesis pathway. HMG-CoA is generated from acetyl-CoA and subsequently converted to mevalonate. Mevalonate undergoes a series of reactions to produce squalene, which is converted to squalene-2,3-epoxide and then cyclized to lanosterol. Cholesterol is synthesized from lanosterol through two interconnected routes, the Bloch and Kandutsch–Russell pathways. In the Bloch pathway, the side-chain double bond is retained; lanosterol undergoes CYP51A1-mediated demethylation to form zymosterol and subsequently desmosterol, which is converted to cholesterol by DHCR24. By contrast, the Kandutsch–Russell pathway involves early reduction of the side-chain double bond; DHCR24 reduces lanosterol to dihydrolanosterol, followed by conversion to zymostenol, lathosterol, and the final intermediate 7-dehydrocholesterol, which is converted to cholesterol by DHCR7. These pathways are not independent, and DHCR24 activity toward multiple intermediates determines the metabolic switch between the Bloch and Kandutsch–Russell pathways. 24S-HC is excreted across the BBB. CDCA also enters the brain from the systemic circulation through the BBB. (**B**) Bile acid biosynthesis in the brain. Cholesterol is converted to CDCA through two major pathways in the central nervous system: the brain-specific pathway and the alternative (acidic) pathway. In the alternative pathway, cholesterol undergoes side-chain oxidation by CYP27A1, 7α-hydroxylation by CYP7B1 to produce 7α,27-dihydroxycholesterol, and it is converted by HSD3B7 to 7α-hydroxy-3-oxo-cholest-4-en-26-oic acid. In the brain-specific pathway, cholesterol is converted to 24S-hydroxycholesterol by CYP46A1, followed by 7α-hydroxylation by CYP39A1 and subsequent conversion by HSD3B7 into 7α-hydroxylated 3-oxo-Δ4 sterol intermediates. Both pathways can ultimately converge on the production of CDCA in the brain. CYP51A1: cytochrome p450 51A1, DHCR24: 24-dehydrocholesterol reductase, FF-MAS: follicular fluid meiosis-activating sterol, DHCR7: 7-dehydrocholesterol reductase, 24S-HC: 24S-hydroxycholesterol, CDCA: chenodeoxycholic acid, BBB: blood–brain barrier, CYP27A1: cytochrome p450 27A1, CYP7B1: cytochrome p450 7B1, HSD3B7: 3β-hydroxy steroid dehydrogenase type 7, CYP46A1: cytochrome p450 46A1, CYP39A1: cytochrome p450 39A1.

**Figure 2:**
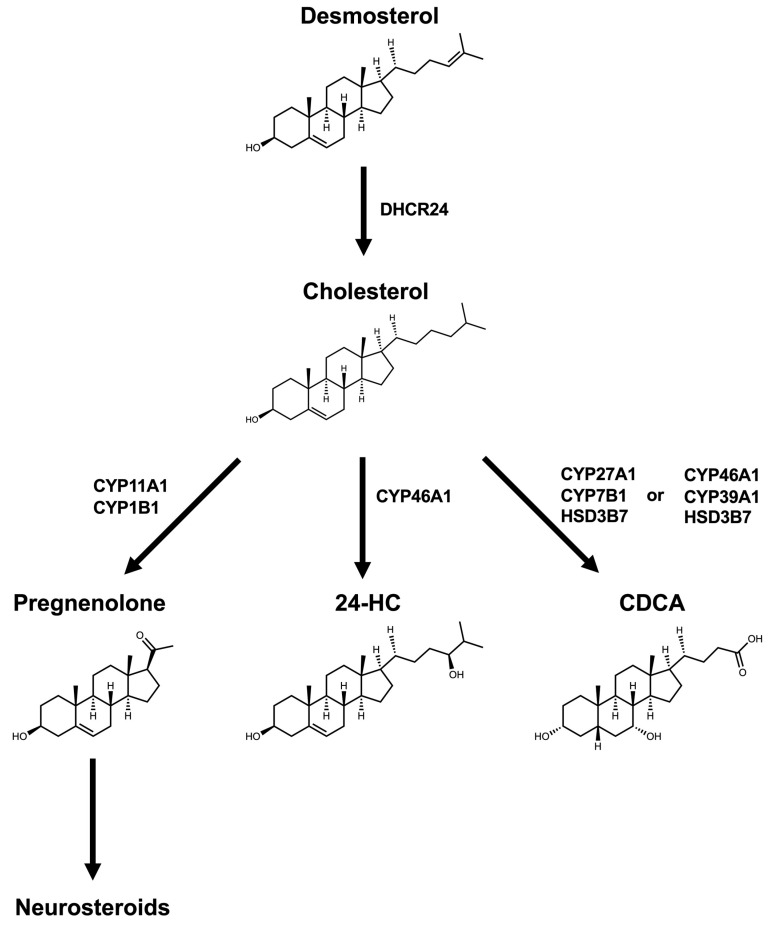
Structures of sterols and their biosynthetic pathways. Desmosterol is converted to cholesterol by DHCR24. Cholesterol is further metabolized to 24S-HC by CYP46A1. CDCA is synthesized via either the alternative (acidic) pathway, mediated by CYP27A1, CYP7B1, and HSD3B7, or the brain-specific pathway, mediated by CYP46A1, CYP39A1, and HSD3B7. In addition, cholesterol is converted by CYP11A1 or CYP1B1 to pregnenolone, which serves as a precursor for multiple neurosteroids. DHCR24: 24-dehydrocholesterol reductase, 24S-HC: 24S-hydroxycholesterol, CYP46A1: cytochrome P450 46A1, CDCA: chenodeoxycholic acid, CYP27A1: cytochrome P450 27A1, CYP7B1: cytochrome P450 7B1, HSD3B7: 3β-hydroxy steroid dehydrogenase type 7, CYP39A1: cytochrome P450 39A11, CYP11A1: cytochrome P450 11A1, CYP1B1: cytochrome P450 1B1.

Cholesterol in the brain is synthesized locally because the brain is separated from peripheral tissues by the BBB, which restricts the entry of cholesterol from the systemic circulation into the brain [5]. Cholesterol synthesis begins with the formation of HMG-CoA from acetyl-CoA (Figure 1A). HMG-CoA is subsequently converted to mevalonate by HMG-CoA reductase, which functions as the rate-limiting enzyme in this pathway. Mevalonate then undergoes a series of phosphorylation and decarboxylation reactions to generate five-carbon isoprenoid units, isopentenyl diphosphate and dimethylallyl pyrophosphate. These intermediates polymerize to form farnesyl pyrophosphate. Two molecules of farnesyl pyrophosphate are then condensed by squalene synthase to produce squalene. Squalene is oxidized by squalene monooxygenase (squalene epoxidase) to form squalene-2,3-epoxide, which is subsequently cyclized by lanosterol synthase to yield lanosterol, the first sterol intermediate containing a steroid nucleus [17,18]. Cholesterol biosynthesis following lanosterol formation involves modification of the sterol nucleus and reduction of the double bond in the side chain at C24. Two biosynthetic routes exist, the Bloch pathway and the Kandutsch–Russell pathway, which differ in the timing of side-chain reduction (Figure 1A). During this process, the steroid core structure is preserved, whereas a subtle structural variation, namely the presence or absence of a double bond at the C24 position of the side chain, determines functional differences between desmosterol and cholesterol. In the Bloch pathway, the double bond is maintained in the side chain. Lanosterol is demethylated by cytochrome P450 51 family subfamily a member 1 (CYP51A1), also known as lanosterol 14α-demethylase, and is subsequently converted to zymosterol and then to desmosterol. Finally, desmosterol is reduced to cholesterol by 24-dehydrocholesterol reductase (DHCR24) (Figure 2). In the Kandutsch–Russell pathway, reduction of the side-chain double bond occurs at an early stage. Lanosterol is reduced by DHCR24, and the pathway proceeds through dihydrolanosterol, zymostenol, and lathosterol. The final intermediate, 7-dehydrocholesterol, is converted to cholesterol by DHCR7. These two pathways are interconnected rather than independent, and DHCR24 activity toward multiple intermediates can result in a metabolic shift from the Bloch pathway to the Kandutsch–Russell pathway [10] (Figure 1A).

#### 2.1.1. Biosynthetic Pathways of Cholesterol in the Brain

The brain is composed of neurons, astrocytes, oligodendrocytes, and microglia [19,20]. Astrocytes and developing neurons preferentially utilize the Bloch pathway for de novo cholesterol synthesis, in which desmosterol serves as the immediate precursor [21]. Although adult neurons employ the Kandutsch–Russell pathway for de novo cholesterol synthesis, this intrinsic synthesis is limited. Instead, cholesterol in the adult brain is mainly synthesized by astrocytes and oligodendrocytes [22]. Moreover, the cholesterol required by adult neurons is supplied predominantly by astrocytes [22]. In neurons, reduced cholesterol levels impair synaptic vesicle exocytosis at the presynapse, leading to inefficient neurotransmitter release [9,23,24]. At the postsynapse, cholesterol contributes to the stability and signal transduction of neurotransmitter receptors, including ionotropic and metabotropic glutamate receptors, as well as GABA receptors [25,26]. Thus, cholesterol is essential for memory formation and consolidation, although neurons largely rely on the uptake of exogenous cholesterol through low-density lipoprotein receptor-related protein 1 to obtain cholesterol from neighboring cells [27,28]. Astrocytes therefore represent the primary source of cholesterol for neurons [22].

#### 2.1.2. Cholesterol Transport in the Brain

Cholesterol synthesized in astrocytes is exported into the extracellular space by ATP-binding cassette (ABC) transporters, such as ABCA1 and ABCG4 [29,30]. Astrocytes also synthesize apolipoprotein E3 (APOE3), which binds cholesterol extracellularly and facilitates its transport to neurons. Astrocytes utilize lipid-free apolipoproteins and lipoproteins to mediate cholesterol efflux [31]. In addition, APOE3 delivers miRNAs to neurons that bind mRNAs involved in cholesterol and sterol biosynthetic processes, such as *HMGCS1*, *HMGCR*, and *CYP51*. This mechanism suppresses de novo cholesterol synthesis and increases intracellular acetyl-CoA levels. The resulting excess acetyl-CoA alters histone acetylation and epigenetically promotes transcription of immediate early genes, such as *Fos*, *Arc*, and *Egr1*, which are associated with neuronal memory [32]. Oligodendrocytes require large amounts of cholesterol for myelin sheath formation. Myelin synthesis depends not only on endogenous cholesterol production by oligodendrocytes but also on cholesterol supplied by astrocytes [33]. Microglia detect and phagocytose surrounding APOE particles and denatured myelin lipids via the triggering receptor expressed on myeloid cells 2, thereby preventing neuroinflammation caused by excessive lipid accumulation [34,35].

In addition, cholesterol biosynthetic intermediates can be converted into other oxysterols. During cholesterol synthesis, squalene is converted to squalene-2,3-epoxide and subsequently to lanosterol; however, at this point, another shunt pathway may occur. Squalene epoxidase converts squalene-2,3-epoxide to 2,3:22,23-dioxidosqualene, which is then converted by lanosterol synthase to 24,25-epoxylanosterol. Following several enzymatic steps, 24,25-epoxycholesterol (24,25-EC) is ultimately produced [36]. Although 24,25-EC is generated in both neurons and astrocytes, astrocytes represent the primary source due to their higher cholesterol biosynthetic capacity [37,38].

### 2.2. Metabolic Conversion to Side-Chain-Retaining Sterols: 24S-HC and CDCA

Cholesterol in the brain is eliminated through its conversion to 24S-HC by CYP46A1 (also known as cholesterol 24-hydroxylase), which is predominantly expressed in neurons rather than astrocytes (Figure 2). Hydroxylation by CYP46A1 increases the hydrophilicity of cholesterol, thereby enabling its excretion across the BBB [22,39,40,41]. Although conversion to 24S-HC is essential for maintaining cholesterol efflux, cholesterol is also further metabolized to generate neurosteroids or CDCA, which are synthesized locally within the brain [42]. Neurosteroid and bile acid synthetic pathways represent distinct branches of brain sterol metabolism, reflecting distinct modes of side-chain modification in the brain.

#### 2.2.1. Neurosteroid Synthesis

Neurosteroids are synthesized in mitochondria, and cholesterol delivery to mitochondria is mediated by steroidogenic acute regulatory proteins (STARs). Specifically, cholesterol transport into mitochondria is facilitated by STARD1 and STARD3. In addition, mitochondria-associated membranes, which form physical contact sites between the endoplasmic reticulum and mitochondria, function as platforms for efficient cholesterol transfer [43]. By contrast, the involvement of mitochondrial translocator protein, which was previously considered essential for mitochondrial cholesterol transport, remains controversial [44,45,46]. Within the inner mitochondrial membrane, CYP11A1 (P450 side chain cleavage, P450scc) converts cholesterol to pregnenolone. Importantly, this reaction involves cleavage of the C27 side chain of cholesterol. Consequently, unlike side-chain-retaining sterols, pregnenolone does not retain the original long aliphatic side chain (Figure 2). Following this cleavage step, pregnenolone serves as a precursor for the synthesis of various neurosteroids in the brain through multiple biosynthetic pathways; however, the presence of CYP11A1 protein in the human brain remains uncertain [47]. Oligodendrocytes have been considered the primary cell type responsible for converting cholesterol to pregnenolone in the brain [48]. However, recent single-cell transcriptome analyses demonstrated that STAR, which mediates cholesterol transport, and Cyp11a1, which catalyzes pregnenolone formation, are primarily co-expressed in neurons in rodents [49]. Therefore, further detailed studies are required to determine the cell type responsible for de novo pregnenolone synthesis. In contrast, CYP1B1 protein, another enzyme capable of cleaving the C27 side chain of cholesterol to produce pregnenolone, has been detected in both human and rodent brains. Moreover, pregnenolone synthesis in human oligodendrocyte glioma cells depends on CYP1B1 activity [50]. Thus, CYP1B1 may represent the enzyme responsible for pregnenolone synthesis in humans. Finally, pregnenolone is further metabolized into diverse neurosteroids through multiple pathways [51,52,53] (Figure 2).

#### 2.2.2. Bile Acid Synthesis

Cholesterol is also converted to bile acids in the brain, which constitute another class of side-chain-retaining sterols [12,54]. As products of sterol metabolism that retain the aliphatic side chain, bile acids are considered alongside 24S-HC as biologically active metabolites with signaling potential in neural tissues. Unlike neurosteroid synthesis, which involves side-chain cleavage, bile acid production proceeds through oxidation and stepwise modification of the side chain while preserving its aliphatic structure. Bile acids are synthesized via two major metabolic routes, the classical (neutral) pathway, regulated by CYP7A1, 3β-hydroxy steroid dehydrogenase type 7 (HSD3B7), and CYP8B1, and the alternative (acidic) pathway, mediated by CYP27A1, CYP7B1, and HSD3B7. Although CYP7A1 functions as the rate-limiting enzyme of the classical pathway in the liver, it is not expressed in the brain. Instead, other enzymes involved in the alternative and brain-specific pathways, such as CYP27A1 and CYP46A1, are expressed in neurons, astrocytes, and oligodendrocytes [49,55,56] (Figure 1B). Thus, bile acid synthesis in the brain relies on alternative pathways rather than the classical pathway [49,54]. In the alternative pathway, cholesterol is first oxidized at the side chain by CYP27A1, generating 27-hydroxycholesterol (27-HC). 27-HC undergoes subsequent 7α-hydroxylation of the steroid nucleus, primarily mediated by CYP7B1, yielding 7α,27-dihydroxycholesterol. The resulting 7α-hydroxylated oxysterols are then converted by HSD3B7, which catalyzes the oxidation of the 3β-hydroxyl group to a 3-oxo group concomitant with isomerization of the Δ5 double bond to a Δ4 position. This reaction converts cholesterol-derived sterols to 7α-hydroxy-3-oxo-cholest-4-en-26-oic acid, transforming them into precursors for bile acids. Subsequent shortening of the side chain can result in the production of CDCA. In addition, bile acid biosynthesis in the brain also occurs through a brain-specific pathway mediated by CYP46A1. Cholesterol is converted to 24S-hydroxycholesterol by CYP46A1, which introduces a hydroxyl group at the C24 position. 24S-hydroxycholesterol undergoes 7α-hydroxylation of the steroid nucleus, primarily attributed to CYP39A1, yielding 7α,24S-dihydroxycholesterol. 7α,24S-dihydroxycholesterol is converted by HSD3B7, which catalyzes the oxidation of the 3β-hydroxyl group to a 3-oxo group concomitant with isomerization of the Δ5 double bond to a Δ4 position. This reaction converts cholesterol-derived sterols into 7α-hydroxylated 3-oxo-Δ4 sterol intermediates, exemplified by 7α-hydroxy-3-oxo-cholest-4-en-24S-ol, thereby committing them to the bile acid biosynthetic pathway. Subsequent shortening of the side chain can ultimately result in the production of CDCA. Thus, the alternative pathway and the brain-specific pathway are capable of generating CDCA in the brain [12,54] (Figure 1B). Bile acids present in the brain originate from either local de novo synthesis or the systemic circulation. Given the observed correlation between cerebral and serum bile acid concentrations, the majority of the brain bile acid pool is thought to derive from the systemic circulation [57,58]. Nevertheless, primary bile acids synthesized locally within the brain may also contribute to physiological and pathophysiological processes.

## 3. Physiological Roles of Side-Chain-Retaining Sterols in the Brain: Desmosterol, 24S-HC, and CDCA

**Figure 3:**
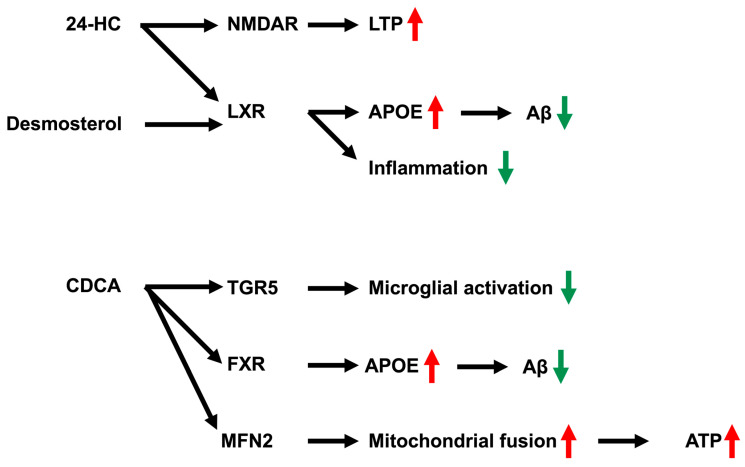
Physiological functions of side-chain-retaining sterols in the brain: desmosterol, 24S-HC, and CDCA. 24S-HC acts as a positive allosteric modulator of NMDA receptors, thereby promoting LTP. Both 24S-HC and desmosterol act as ligands for LXRs. LXR activation upregulates APOE expression, facilitates Aβ clearance, and suppresses inflammatory responses. CDCA functions as a ligand for FXR and TGR5. FXR activation induces APOE expression, potentially enhancing Aβ clearance in the brain. TGR5 activation in microglia suppresses the production of inflammatory cytokines. In addition, CDCA directly binds to and activates MFN2, promoting mitochondrial fusion and increasing ATP production. 24S-HC: 24S-hydroxycholesterol, NMDAR: N-methyl-D-aspartate receptor, LTP: long-term potentiation, LXR: liver X-receptor, APOE: apolipoprotein E, Aβ: amyloid-beta peptide, CDCA: chenodeoxycholic acid, TGR5: Takeda G protein-coupled receptor 5, FXR: farnesoid X receptor, MFN2: mitofusin 2.

Side-chain-retaining sterols (desmosterol, 24S-HC, and CDCA) retain their characteristic aliphatic side chains. These sterols exert genomic effects through nuclear receptors, resulting in functional changes mediated by transcription and protein synthesis and thereby contributing to long-term and broad-based regulation. In addition, these sterols exert nongenomic effects through membrane receptors localized near synapses, allowing rapid modulation of neural activity. Accordingly, side-chain-retaining sterols in the brain participate in both local, immediate regulation at synapses and global, long-term regulation at cell bodies (Figure 3). The effects of these side-chain-retaining sterols are not limited to a single cell, but rather may contribute to metabolic crosstalk between neurons and glial cells through cell type-specific production of side-chain-retaining sterols and their receptor expression.

### 3.1. Roles of the Side-Chain-Retaining Cholesterol Precursor Desmosterol in the Brain

Desmosterol accumulates during brain development [6]. Excess cholesterol in the brain is converted to 24S-hydroxycholesterol and subsequently excreted; however, desmosterol is not hydroxylated for excretion. As a result, desmosterol is maintained in a form that limits its removal from the brain and promotes its accumulation. Once cholesterol is esterified, it becomes unavailable for membrane construction. In contrast, desmosterol exhibits lower esterification efficiency than cholesterol, thereby inhibiting cholesterol esterification. These mechanisms enable cholesterol, a membrane component essential for brain development, to be retained without excessive excretion. Because astrocytes are the primary source of cholesterol for neurons [22], the cholesterol precursor desmosterol is also likely synthesized predominantly in astrocytes. Moreover, desmosterol functions as a potent agonist of LXR [59,60,61,62,63]. LXR activation increases the expression of APOE and ABCA1, thereby facilitating cholesterol delivery to neurons required for synapse formation and neuronal growth [64]. In addition, APOE plays an important role in the clearance of amyloid beta peptide (Aβ), a major contributor to Alzheimer’s disease pathology [65]. By contrast, genetic deficiency of DHCR24, which converts desmosterol to cholesterol, leads to persistent desmosterol accumulation and causes desmosterolosis, a disorder characterized by brain malformations and additional symptoms [66,67]. Thus, proper regulation of desmosterol levels in the brain is essential for normal brain development.

Desmosterol and 24S-HC both serve as ligands for LXR [59,60,61,62,63]. Here, LXR-mediated signaling primarily regulates cholesterol transport and inflammatory gene expression, distinguishing it from other receptor pathways discussed below. LXR activation suppresses the expression of proinflammatory genes, resulting in reduced inflammatory responses [68]. Upon ligand binding, LXR undergoes SUMOylation and inhibits the dissociation of the nuclear receptor corepressor from inflammatory gene promoters, thereby suppressing inflammation [69,70]. Furthermore, activated LXR binds enhancer regions of inflammatory genes, induces chromatin compaction, and represses inflammatory gene expression [71]. Through these mechanisms, desmosterol may suppress neuroinflammation, which is considered a contributing factor to neurodegenerative diseases, and may therefore participate in neuroprotection. In addition, desmosterol acts as an agonist for retinoic acid-related orphan receptor γ (RORγ) [72]. RORγ is a key regulator of circadian rhythms [73,74].

### 3.2. Roles of Side-Chain-Retaining Sterols 24S-HC and CDCA in the Brain

24S-HC is a highly abundant side-chain-retaining oxysterol in the brain that is primarily synthesized by the neuron-specific enzyme CYP46A1. Although 24S-HC is an excretory product, it also functions as a signaling molecule in the brain. 24S-HC acts as an agonist of LXR. Neuron-derived 24S-HC activates LXR in astrocytes and induces the expression of lipid transport-related genes, including APOE and ATP-binding cassette transporters (ABCA1 and ABCG1) [64]. In addition, LXR activation induces *APOE* transcription, which mediates Aβ clearance and lipid transport to regions required for synapse formation and neuronal growth [8,65]. These findings suggest that neuronally produced 24S-hydroxycholesterol regulates lipid transport-related gene expression and amyloid beta peptide clearance via LXR signaling in glial cells, thereby indirectly promoting neuroprotection. In contrast, 24S-HC also acts as a positive allosteric modulator of N-methyl-D-aspartate (NMDA) receptors at synapses. Notably, NMDA receptor modulation represents a synaptic mechanism distinct from the transcriptional regulation mediated by nuclear receptors such as LXR. By increasing the probability of NMDA channel opening, 24S-HC enhances calcium influx and long-term potentiation [75,76]. In addition, 24S-HC functions as an agonist for RORγ [77]. Therefore, 24S-HC may be related to circadian rhythm regulation.

CDCA is another side-chain-retaining sterol synthesized in the brain through the alternative pathway, mediated by CYP27A1, CYP7B1, and HSD3B7, as well as through a brain-specific pathway mediated by CYP46A1, CYP39A1, and HSD3B7. CDCA serves as a natural ligand for the nuclear receptor FXR. In contrast to LXR signaling, FXR activation primarily regulates bile acid–related metabolic pathways and lipid homeostasis. Activation of FXR induces APOE expression [14]. It not only prevents excessive lipid accumulation within cells but also promotes the clearance of Aβ from the brain, which are key components of Alzheimer’s disease pathology. In contrast, CDCA also acts as an agonist for the membrane receptor Takeda G protein-coupled receptor 5 (TGR5). TGR5 couples to Gs proteins, increases intracellular cAMP levels, activates protein kinase A, and stimulates exchange proteins directly activated by cAMP [78]. Furthermore, TGR5 activation inhibits NF-κB signaling, resulting in suppression of proinflammatory cytokine release, including TNF-α and IL-1β [79,80]. In microglia, TGR5 activation leads to reduced production of proinflammatory cytokines [81]. Thus, TGR5 signaling represents a membrane receptor–mediated pathway that modulates inflammatory responses independently of nuclear receptor signaling. In addition, CDCA directly binds to and activates MFN2, a key regulator of mitochondrial architecture, thereby promoting mitochondrial fusion and increasing ATP production [82]. Importantly, these sterols activate distinct receptors in different neural cell types, including nuclear receptors such as LXR and FXR, membrane receptors such as TGR5, and synaptic NMDA receptors, each of which mediates specific downstream effects.

## 4. Neurodegenerative Diseases and Side-Chain-Retaining Sterols

**Figure 4:**
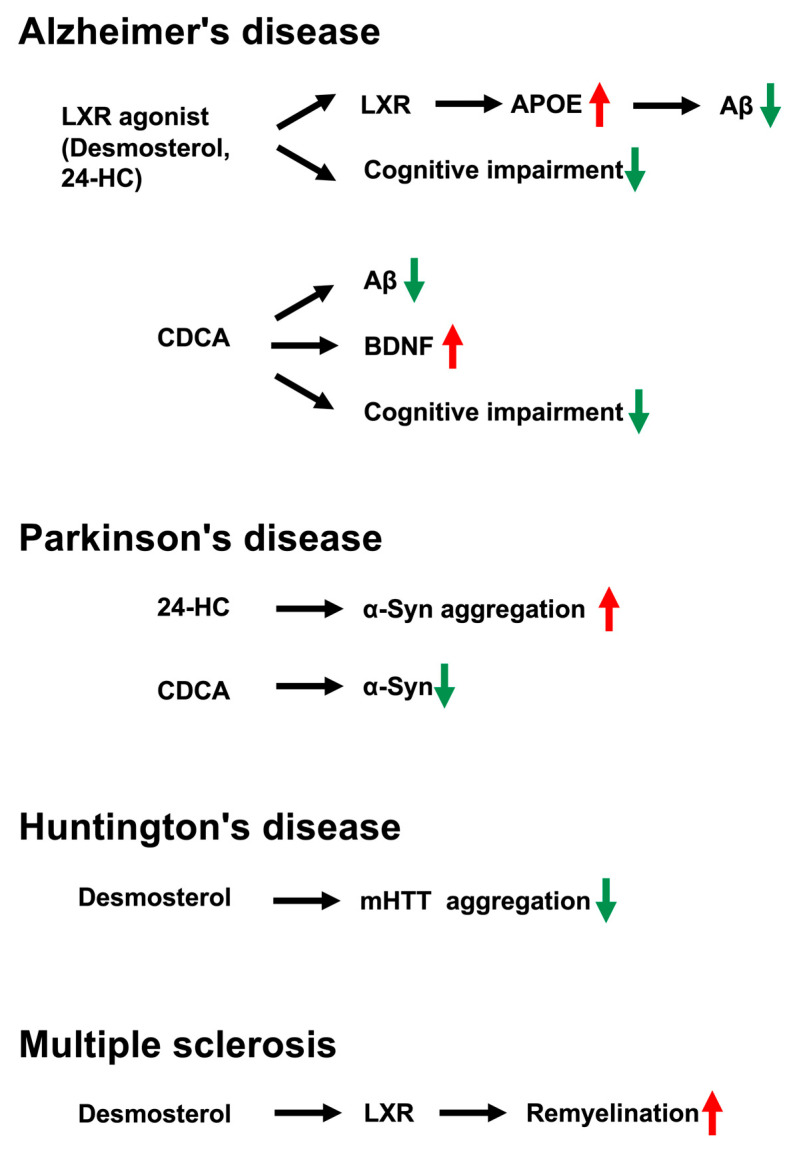
Neurodegenerative diseases and side-chain-retaining sterols. Alzheimer’s disease: LXR agonists upregulate the expression of APOE, ABCA1, and AQP4, thereby reducing Aβ levels and reversing cognitive decline in Alzheimer’s disease models. As LXR ligands, desmosterol and 24S-HC may exert similar neuroprotective effects. CDCA improves cognitive function and reduces Aβ levels, and also increases BDNF expression. Parkinson’s disease: 24S-HC promotes aggregation of α-Syn, leading to fibril formation with enhanced seeding activity and increased neurotoxicity. By contrast, CDCA suppresses elevations in α-Syn levels, potentially exerting protective effects against α-synucleinopathy. Huntington’s disease: Desmosterol significantly reduces mHTT aggregate formation, suggesting a protective role in Huntington’s disease pathology. Multiple sclerosis: Repair of inflamed demyelinated lesions requires microglial clearance of myelin debris and resolution of inflammation. Microglia-derived desmosterol activates LXR signaling, which suppresses neuroinflammation and promotes remyelination by oligodendrocytes. LXR: liver X-receptor, 24S-HC: 24S-hydroxycholesterol, APOE: apolipoprotein E, Aβ: amyloid-beta peptide, CDCA: chenodeoxycholic acid, BDNF: brain-derived neurotrophic factor, α-Syn: α-synuclein, mHTT: mutant huntingtin.

In neurodegenerative diseases, the actions of side-chain-retaining sterols are mediated through receptor-specific pathways, including LXR- and FXR-dependent transcriptional regulation as well as membrane receptor–mediated signaling. Alzheimer’s disease is a progressive neurodegenerative disorder characterized by memory impairment, dementia, and morphological alterations in the brain. Its pathological hallmarks include the accumulation of Aβ and aggregation of tau protein. In particular, soluble Aβ oligomers, generated through aggregation of Aβ monomers, exert strong synaptic toxicity and are considered a primary cause of synaptic dysfunction preceding neuronal loss. Aβ is produced by proteolytic processing of amyloid precursor protein by β- and γ-secretases [83,84]. Desmosterol levels are reduced in patients with Alzheimer’s disease [85]. CDCA improved cognitive performance and reduced beta-site amyloid precursor protein-cleaving enzyme 1 and Aβ levels in an Alzheimer’s disease rat model treated with AlCl_3_. Moreover, CDCA activated cAMP response element-binding protein (CREB) and increased brain-derived neurotrophic factor (BDNF) levels in the hippocampus of an Alzheimer’s disease rat model [86] (Figure 4). In contrast, Aβ-induced apoptosis enhanced FXR expression in differentiated SH-SY5Y cells and mouse hippocampal neurons. Treatment with 6ECDCA, an FXR ligand, further enhanced Aβ-induced neuronal apoptosis, accompanied by reduced CREB activity and decreased BDNF expression [87]. CREB activation induces expression of BDNF and the antiapoptotic protein Bcl2 [88]. Therefore, CDCA putatively enhances Aβ-induced neuronal apoptosis through FXR signaling. In contrast, LXR agonists, such as GW3965 and TO901317, upregulate APOE and ABCA1 expression, reduce Aβ levels, and reverse cognitive decline in mouse models of Alzheimer’s disease [89,90,91,92,93]. In addition, CE9A215, a compound extracted from chaga mushroom, acts as an LXR ligand and regulates expression of APOE, ABCA1, and aquaporin-4 in the brain through LXR activation. CE9A215 also suppresses cognitive decline, reduces Aβ accumulation, and attenuates neuroinflammation in the hippocampus of an Alzheimer’s disease mouse model [94]. Thus, because side-chain-retaining sterols function as LXR ligands, they may exert similar modulatory effects in Alzheimer’s disease.

Parkinson’s disease is a progressive neurodegenerative disorder characterized by tremors, muscle rigidity, impaired voluntary movement, bradykinesia, and postural instability. Pathologically, dopaminergic neurons in the nigrostriatal pathway of the midbrain are lost, and insoluble intracellular aggregates composed primarily of α-synuclein (α-Syn) accumulate within neurons [95]. CDCA attenuated motor deficits, improved anxiety-like behavior, exhibited antidepressant effects, and improved cognitive function in a mouse model of 1-methyl-4-phenyl-1,2,3,6-tetrahydropyridine (MPTP)-induced Parkinson’s disease. Furthermore, CDCA improved the morphological and histological characteristics of brain neurons and inhibited MPTP-induced dopaminergic degeneration. CDCA also suppressed the increase in α-Syn levels (Figure 4). Collectively, it putatively exerts a protective function against Parkinson’s disease [96]. The LXR agonist TO901317 prevented dopaminergic neuron deterioration and improved locomotor activity in an MPTP-treated mouse model of Parkinson’s disease [97]. Levels of 24S-HC were correlated with cerebrospinal fluid parameters in patients with Parkinson’s disease [98,99]. In contrast, 24S-HC promotes α-Syn aggregation, which results in the formation of α-Syn fibrils with increased seeding activity, neurotoxicity, and neurodegeneration. Pre-formed α-Syn fibrils generated in the presence of 24S-HC exhibited greater seeding potency than control fibrils [99] (Figure 4). Thus, 24S-HC may facilitate neurotoxicity, α-Syn propagation, and Parkinson’s disease progression.

Huntington’s disease is characterized by motor dysfunction, cognitive decline, and psychiatric disturbances. It is an autosomal dominant neurodegenerative disorder caused by mutations resulting in polyglutamine expansion at the N-terminus of huntingtin (HTT), leading to progressive and significant atrophy of the striatum and cerebral cortex [100,101,102]. Desmosterol significantly reduced mutant huntingtin (mHTT) aggregate formation in mouse striatal neurons expressing mHTT [103] (Figure 4).

Amyotrophic lateral sclerosis (ALS) is a neurodegenerative disease that causes bulbar paralysis and progressive loss of limb function. Patients with ALS usually die from respiratory failure within three years of symptom onset [104]. In a cohort study including 438 patients with ALS and 330 healthy controls, two LXR single-nucleotide polymorphisms were associated with age at disease onset. These findings suggest that LXR signaling may contribute to ALS progression or to the maintenance of central nervous system viability. However, the effects of LXR ligands in ALS remain unclear, and further investigation is required [105].

Multiple sclerosis is a multifactorial autoimmune demyelinating and neurodegenerative disease characterized by central nervous system inflammation, demyelination, and axonal loss, accompanied by attempted remyelination by oligodendrocytes [106]. Repair of inflamed demyelinated lesions requires the removal of myelin debris by microglia and the suppression of inflammatory lesions. Microglia synthesize desmosterol, which activates LXR signaling to suppress inflammation and promote remyelination by oligodendrocytes [107] (Figure 4). Other side-chain-retaining sterols that act as LXR ligands may similarly exert anti-inflammatory effects in multiple sclerosis.

Side-chain-retaining sterols act as key endogenous regulators in various neurodegenerative diseases. They influence disease processes by modulating receptor-dependent transcriptional programs, inflammatory responses, mitochondrial function, and protein aggregation pathways. Through their multifaceted roles as receptor ligands, they may play a crucial part in maintaining the balance between physiological functions and neurodegeneration within the brain.

## 5. Concluding Remarks

The BBB allows the brain to synthesize cholesterol independently of the systemic circulation, thereby maintaining a central cholesterol pool. Although the brain accounts for only 2% of total body weight, it contains approximately 23% of the body’s total cholesterol. Regulation of this cholesterol pool is not limited to structural maintenance, because cholesterol precursors, cholesterol itself, and cholesterol metabolites function as physiologically active molecules. While multiple cholesterol precursors and metabolites are present in the brain, dedicated regulatory mechanisms exist for side-chain-retaining sterols, including cholesterol precursors, oxysterols, and bile acids. These molecules act as ligands for specific receptors to modulate cellular responses. Side-chain-retaining sterols operate through dual regulatory mechanisms, comprising a genomic pathway mediated by nuclear receptors and a nongenomic pathway mediated by membrane-bound receptors. Genomic pathways regulate expression of proteins such as APOE, which is involved in lipid transport, as well as cytokines associated with inflammatory responses, through nuclear receptors such as LXR. Nongenomic pathways act through interactions with membrane-bound receptors, including NMDA receptors, enabling rapid physiological responses. Together, these complementary regulatory mechanisms coordinate long-term cellular homeostasis with immediate synaptic and inflammatory responses. The roles of side-chain-retaining sterols in the brain further suggest their involvement in functional coupling and metabolic crosstalk between neurons and glial cells. Moreover, side-chain-retaining sterols represent important modulators in a range of neurodegenerative diseases. Neurodegenerative diseases and neuroinflammation are closely related. An imbalance in side-chain-retaining sterols may alter the activity of nuclear receptors in microglia and astrocytes, affecting chronic inflammatory gene expression. Therefore, side-chain sterols and their related substances that can cross the BBB, or drugs that can regulate the abundance of side-chain-retaining sterols, may be promising therapeutic strategies for suppressing chronic inflammation in the brain. Although evidence supporting the physiological functions and pathological relevance of side-chain-retaining sterols in neurodegenerative diseases is steadily accumulating, detailed understanding of the cell type-specific regulation and molecular mechanisms underlying side-chain-retaining sterol signaling in the brain remains limited. This review emphasizes that side-chain-retaining sterols present in the brain are not merely intermediates or excretory products of cholesterol metabolism but endogenous signaling molecules that are functionally distinct according to their biosynthetic pathways and structural properties. Through these properties, side-chain-retaining sterols may influence brain function and neurodegenerative disease processes. Further clarification of the molecular mechanisms governing their production and actions is expected to advance understanding of the therapeutic potential of side-chain-retaining sterols.

## Data Availability

No new data were created or analyzed in this study.
